# The Role of Artificial Intelligence in Orthognathic Surgery: A Scoping Review

**DOI:** 10.3390/dj14050286

**Published:** 2026-05-11

**Authors:** Katarína Janáková, Barbora Heribanová, Juraj Tomášik, Daniela Tichá, Martin Strunga, Andrej Janák, Kristián Šimko, Andrej Thurzo

**Affiliations:** 1 Department of Orthodontics, Regenerative and Forensic Dentistry, Faculty of Medicine and KORFS Comenius University in Bratislava, Dvořákovo Nábrežie 4, 81102 Bratislava, Slovakia; heribanova24@uniba.sk (B.H.); tomasik7@uniba.sk (J.T.); ticha46@uniba.sk (D.T.); strunga2@uniba.sk (M.S.); thurzo3@uniba.sk (A.T.); 2 Urological Clinic of University Hospital in Bratislava, Faculty of Medicine, Comenius University in Bratislava, Ružinovská 6, 82606 Bratislava, Slovakia; janak14@uniba.sk; 3 Department of Oral and Maxillofacial Surgery, Faculty of Medicine, University Hospital Bratislava, Comenius University, Ružinovská 6, 82606 Bratislava, Slovakia

**Keywords:** AI, virtual surgical planning, machine learning, deep learning, cephalometric analysis, dentofacial deformities, surgical outcome prediction

## Abstract

**Background/Objectives:** Artificial intelligence (AI) has gained growing interest in the field of orthognathic surgery due to its potential to improve diagnostic accuracy, surgical planning, and treatment outcomes. This scoping review maps literature from 2017 to May 2025 to identify AI applications in orthognathic surgery, assess their clinical relevance, and discuss the associated ethical, legal, and technical limitations. **Methods:** This scoping review further examines the stages of the orthognathic surgical workflow at which AI applications have been prospectively validated, the artificial intelligence methodologies applied to virtual surgical planning and outcome prediction, and the main methodological, ethical, and legal factors that may constrain broader clinical adoption. **Results:** A total of 62 studies were included, covering AI use in cephalometric analysis, virtual surgical planning (VSP), outcome prediction, and intraoperative support. While AI demonstrates remarkable potential in orthognathic planning, current approaches are often limited by heterogeneous methodologies and retrospective validation. **Conclusions:** Future studies should prioritize prospective, multicentre designs integrating AI-assisted decision-making directly into the clinical workflow, with emphasis on model interpretability, patient-specific accuracy, and ethical transparency. These questions extend beyond mapping applications by emphasizing clinical validation, methodological rigor, and ethical accountability—dimensions insufficiently explored in prior reviews.

## 1. Introduction

Orthognathic surgery is an interdisciplinary field involving orthodontics and maxillofacial surgery, focused on correcting skeletal and functional jaw anomalies, primarily caused by abnormal development, in cases where orthodontic treatment alone is insufficient. This surgical approach, together with orthodontic preparation and follow-up treatment, significantly impacts facial aesthetics, reestablishes dental occlusion, and enhances overall patient quality of life and self-esteem. Orthognathic treatment planning involves a clinical examination, assessment of occlusal relationships, imaging studies, cephalometric analysis, and orthodontic preparation. However, with the introduction of advanced digital technologies and the planning and execution of orthognathic procedures have become increasingly more accurate and individualized [1,2].

Artificial intelligence (AI) is defined as a ’system’s ability to correctly interpret external data, learn from such data, and to use those learnings to achieve specific goals and tasks through flexible adaptation’ [3]. Recent advances in computer science and informatics have made AI an increasingly integral component of modern healthcare, including surgical disciplines. AI-driven algorithms and applications increasingly assist medical professionals in clinical practice and contribute to medical research [4]. Dentistry has always benefited from technological advances in medicine and healthcare. Within Orthodontics, recent research succeeded in demonstrating that AI is the most studied digital technology, more than half of recent highly cited papers pertain to AI [5,6]. With AI algorithms being increasingly developed, researchers try to challenge it and show whether its implementation in orthodontic diagnosis and treatment planning is both helpful and accurate. In 2024, it was shown that automated tracing of 3D cephalometric images obtained from CBCT was slower and less precise than tracing performed by skilled clinicians [7]. Nowadays, on the other hand, it seems that even freely accessible AI tools could suggest which facial changes are desirable in a patient and what an orthodontist should aim for [8]. Similarly, in orthognathic surgery, AI is utilized for imaging analysis (CBCT, MRI, 3D scans), virtual surgical planning, automated outcome prediction, and personalized treatment strategies based on parameters such as Angle’s classification I for dental occlusion and cephalometric values for skeletal assessment. Its implementation may improve precision, efficiency, and predictability in surgical procedures. It can also help reduce treatment errors, which may lead to better clinical outcomes and patient care [9].

The aim of this scoping review is to systematically map and summarize current knowledge on the applications of AI in orthognathic surgery. This study focuses on identifying key areas where AI provides the most significant benefits, analysing its advantages and limitations, and discussing potential future developments. To achieve this, a scoping review of the available literature was conducted, with an emphasis on relevant clinical applications and technological innovations.

## 2. Materials and Methods

### 2.1. Study Design and Reporting Framework

This scoping review followed the PRISMA-ScR (Preferred Reporting Items for Systematic Reviews and Meta-Analyses Extension for Scoping Reviews) guidelines.

### 2.2. Protocol Registration

The review protocol was registered on the Open Science Framework (OSF) (https://osf.io/x3w2s) (accessed on 7 May 2026).

### 2.3. Information Sources and Search Strategy

A systematic literature search was performed in PubMed, Scopus, and Web of Science for articles published between January 2017 and May 2025. The literature search was conducted between March 2025 and September 2025. The following search query was applied: (“Artificial intelligence” OR “Machine learning”) AND (“Orthognathic surgery” OR “Virtual surgical planning” OR “Jaw surgery” OR “Maxillofacial surgery” OR “Outcome prediction”).

### 2.4. Eligibility Criteria

#### 2.4.1. Inclusion Criteria

Literature was included if they were peer-reviewed, published in English, and focused on AI applications in orthognathic surgery, specifically in the areas of imaging analysis, virtual surgical planning, intraoperative assistance, or outcome prediction.

#### 2.4.2. Exclusion Criteria

Excluded were case reports, editorials, and purely technical algorithm papers without surgical relevance and studies not specific to orthognathic surgery.

### 2.5. Charting of Data

Data extraction and synthesis were conducted using a structured matrix to compare study design, AI model types, data sources (CBCT, radiographs, clinical photographs), evaluation metrics, and reported clinical outcomes. In addition to thematic synthesis, detailed study-level data including AI algorithms, sample sizes, validation strategies, and performance metrics were systematically extracted and are provided in Supplementary Table S1.

### 2.6. Collating, Summarizing and Reporting of the Results

Thematic synthesis was then applied to summarize key advantages, limitations, and emerging trends in AI integration within orthognathic surgery.

### 2.7. Quality Appraisal of Included Primary Studies

As this study followed a scoping review methodology, a formal risk-of-bias assessment was not performed. A formal appraisal of study quality or level of evidence (e.g., Oxford Centre for Evidence-Based Medicine—OCEMB levels) was not performed, as this review followed a scoping review methodology aimed at mapping the extent and nature of available evidence rather than assessing methodological quality.

## 3. Results

**Figure 1:**
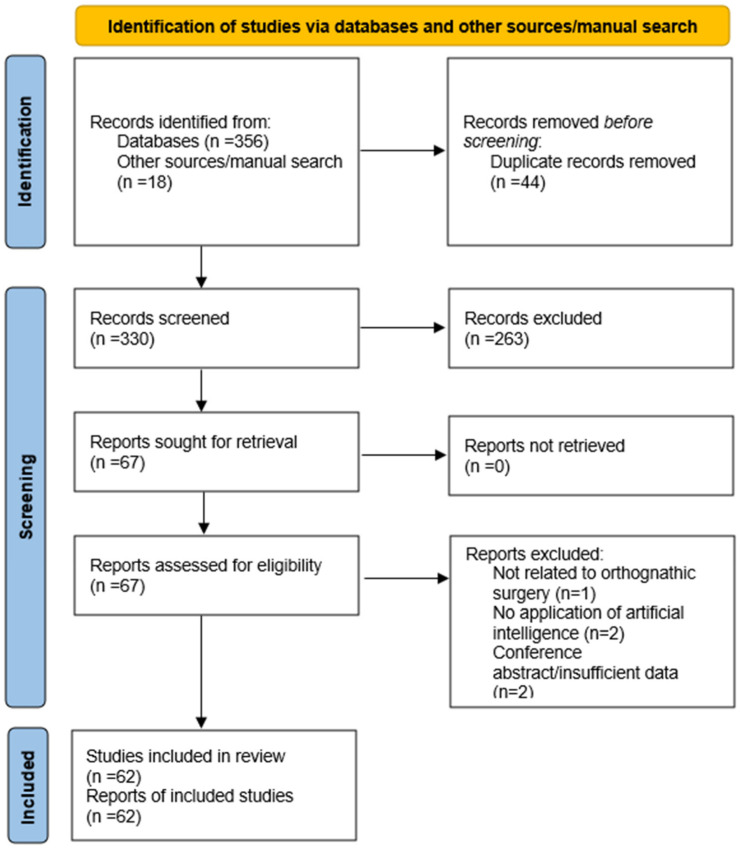
PRISMA 2020 flow diagram illustrating the study selection process.

A total of 374 records were identified through database searching and other sources (PubMed: *n* = 135; Scopus: *n* = 112; Web of Science: *n* = 109; manual search: *n* = 18). After removal of duplicates (*n* = 44), 330 records remained for title and abstract screening. Following this step, 67 full-text articles were assessed for eligibility. After full-text evaluation, 62 studies met the inclusion criteria and were subsequently included in the qualitative synthesis, as illustrated in Figure 1.

Data extraction focused on artificial intelligence methodology, area of application (including cephalometric analysis, virtual surgical planning, intraoperative assistance, and outcome prediction), reported outcomes (such as diagnostic accuracy, surgical efficiency, and predictive performance), as well as methodological quality attributes, including study design, sample size, and transparency of reporting.

**Figure 2:**
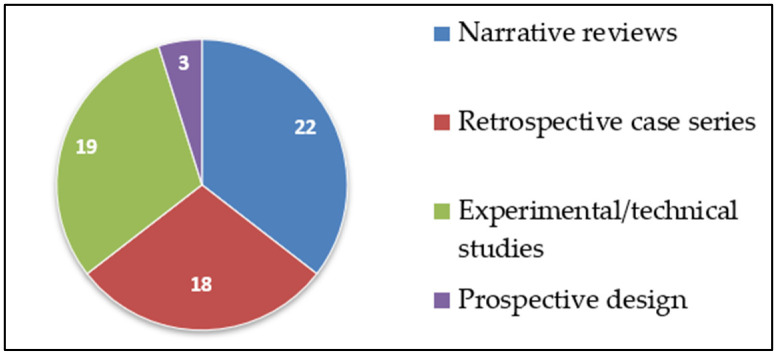
Distribution of included studies by study design.

As illustrated in Figure 2, most of the included studies consisted of narrative or scoping reviews (*n* = 22), followed by experimental or technical studies (*n* = 19) and retrospective observational or case series studies (*n* = 18). Only a limited number of studies employed a prospective design (*n* = 3).

A substantial proportion of the included literature consisted of narrative and scoping reviews (*n* = 22). These secondary studies were included to provide an overview of prevailing research directions, methodological trends, and conceptual frameworks related to the application of artificial intelligence in orthognathic surgery. However, their inclusion may introduce a degree of redundancy, as several reviews summarize overlapping sets of primary studies. For this reason, narrative reviews were not weighted as primary evidence when interpreting methodological maturity or clinical validation but were instead used to contextualize findings derived from original research studies.

Common methodological limitations across the included literature included selection bias, small sample sizes, limited external validation, and a lack of transparency regarding AI model development and validation procedures. Several studies did not clearly report the datasets used for model training or testing, and only a minority provided access to publicly available data, thereby limiting reproducibility and generalizability of the findings.

### Summary Table of Included Studies

Given the breadth and heterogeneity of the included literature (*n* = 62), Table 1 presents a structured thematic overview of key application areas of artificial intelligence in orthognathic surgery, rather than an exhaustive listing of individual studies. To enhance transparency and illustrate methodological heterogeneity across individual studies, a comprehensive study-level data extraction table is provided as Supplementary Table S1.

The studies were grouped according to their primary clinical application (cephalometric analysis, virtual surgical planning, outcome prediction, intraoperative assistance, ethical and policy considerations, and general AI concepts), with corresponding study types, key findings, and representative references provided for each category.

This approach enabled a concise yet comprehensive synthesis of the main research directions, methodological characteristics, and clinical implications of AI applications in orthognathic surgery, while accommodating the diversity in study design and scope across the included literature.

## 4. Discussion

Artificial intelligence has been increasingly utilized across several aspects of orthognathic surgery, ranging from diagnosis and planning to outcome prediction. The limited number of prospective studies identified in this review suggests that the application of artificial intelligence in orthognathic surgery is still at an early stage of development. The included studies demonstrated varying levels of methodological rigor and clinical applicability.

### 4.1. Application of Artificial Intelligence in Orthognathic Surgery

AI has had a major impact on orthognathic surgery, driving advancements in preoperative planning, surgical precision, and postoperative evaluation. AI-powered technologies enhance diagnostic accuracy, optimize surgical workflows, and improve patient outcomes. This technology utilizes machine learning. It enables computers to process data and records, recognize specific patterns, and use them to generate valuable information for specialists [24,31,32].

#### 4.1.1. AI in Imaging and Diagnosis

One of the most essential contributions of AI in orthognathic surgery is its ability to analyse medical images such as X-rays, CT scans, cone-beam computed tomography (CBCT) scans, cephalometric radiographs, and 3D facial imaging with high precision, and subsequently detect skeletal discrepancies, asymmetries, and occlusal abnormalities based on deep learning models, particularly convolutional neural networks (CNNs), which have been shown to outperform traditional manual analysis, thus speeding up the diagnostic process [33,34]. In this context, recent studies have demonstrated that smartphone-based 3D facial scanning applications utilizing TrueDepth technology can achieve high accuracy when compared to CBCT, with mean surface deviations below 0.4 mm, supporting their integration into digital diagnostic workflows and virtual patient creation [35]. Importantly, when combined with artificial intelligence-based processing, such facial scan data can be used for automated landmark detection, surface registration, and integration with CBCT-derived skeletal models, enabling their application in orthognathic surgical planning, outcome simulation, and postoperative soft-tissue assessment rather than serving solely as an image acquisition tool [35]. AI-powered imaging tools provide more detailed 3D visualizations of the operative field, which may assist surgeons in achieving more precise planning and potentially improve surgical outcomes. Although AI shows significant potential, additional studies and validation are still required in challenging cases [20,33,36,37,38,39].

AI-based cephalometric analysis has significantly reduced variation in interpretations among different evaluators and enhanced precision in detecting anatomical reference points (landmarks), which is crucial for treatment planning. These AI models can predict post-surgical facial changes based on preoperative imaging, allowing for individualized treatment approaches [10,40,41,42].

AI can identify anatomical structures with accuracy comparable to experts while significantly reducing the time required compared to manual landmark annotations where human error can be present [43]. It marks these reference points with high precision, even in images with foreign body, such as surgical plates, screws, fixed retainers or brackets [12,13,14]. However, early investigations into AI-assisted 3D cephalometric tracing have revealed that automated landmark detection may still be less accurate and slower than manual tracing by experienced clinicians. Deep learning (DL) has the capability to recognize complex patterns within datasets and can be applied to various imaging modalities, such as posterior radiographs, to enhance diagnostic accuracy in orthognathic surgery. AI can also determine key anatomical features that indicate the need for surgical intervention and by analysing radiographic images, AI can either use anatomical structures as input data or highlight relevant regions of interest to improve outcomes. AI can also analyse facial photographs and accurately identify patients who may require orthognathic surgery [22,44,45,46,47].

#### 4.1.2. AI-Driven Virtual Surgical Planning (VSP)

Virtual surgical planning (VSP) has become an essential tool in orthognathic surgery, enabling surgeons to simulate surgical movements through computer-aided workflows, predict treatment outcomes, design personalized surgical and orthodontic appliances using CAD/CAM technology, and customize procedures based on patient-specific anatomical data derived from CBCT scans. While CBCT-based datasets provide detailed static anatomical information, comprehensive virtual surgical planning increasingly benefits from the integration of additional patient-specific data to achieve truly individualized treatment strategies [18,33,48]. Digital jaw-tracking systems such as Cadiax^®^ 2 and Modjaw^®^ have demonstrated high intra-session and inter-session reliability in recording condylar parameters, including sagittal condylar inclination and Bennett angles, with intraclass correlation coefficients exceeding 0.90, supporting their incorporation into advanced digital and AI-assisted planning workflows [49]. Machine learning algorithms enhance 3D modelling and simulation accuracy, improving surgical precision and reducing the risk of errors which often lead to increased costs and time [50,51].

AI has been used in 3 steps:

- AI-assisted segmentation of CBCT scans enables automated identification of anatomical structures, such as the mandible, maxilla, and temporomandibular joint (TMJ), which facilitates more efficient preoperative planning [14].
- Deep learning models can predict optimal osteotomy sites and movement vectors, improving the alignment of bone segments, reducing operative time and minimizing the risk of complications, such as bone contact problems or misaligned rotations during surgery. Computer-aided manufacturing of surgical splints also improves surgical precision [40].
- AI-driven occlusion simulation helps predict postoperative occlusion stability, reducing the need for revisions and secondary procedures [20].

Accurate pre-surgical planning is essential for the success of orthognathic surgery in correcting dentofacial deformities, with orthodontic treatment playing a crucial role in this process. Two main surgical concepts are currently recognized: the surgery-first approach and the orthodontic decompensation-first approach. In the latter, by the end of orthodontic preparation, the patient’s occlusion should be decompensated to align dental and skeletal discrepancies across all planes while eliminating any interferences that could compromise the final occlusion. At the same time, resolving tooth size discrepancies is necessary to ensure a stable postoperative overjet and overbite [16,20,33,40]. This applies to the procedure—orthodontic decompensation first, but in some cases, AI can simulate the final occlusion without prior orthodontic treatment. If this occlusion is stable, surgery first may be indicated.

#### 4.1.3. Intraoperative—Surgical Procedure

Although computer-aided design/manufacturing (CAD/CAM) systems and three-dimensional (3D) printing technologies are not artificial intelligence (AI) methods per se, several included studies described workflows in which these conventional digital tools were combined with AI-driven components. In such cases, artificial intelligence was primarily applied in upstream stages, including automated segmentation, predictive modeling, decision support, or optimization of surgical planning, while CAD/CAM and 3D printing served as execution tools for translating AI-assisted plans into physical surgical guides and fixation devices.

After the virtual surgical simulation phase, the next step involves designing the occlusal splint and creating surgical guides for osteotomy placement, offering greater precision and efficiency compared to traditional manual methods. More recently, CAD/CAM technology has enabled the development of patient-specific fixation systems [17,18,52,53]. Studies evaluating digital workflows have demonstrated that computer-based design and manufacturing processes can significantly reduce chairside time and overall procedural costs compared to conventional techniques, highlighting the efficiency benefits of digitally planned and fabricated surgical components [54]. Once designed, the occlusal splint data is exported and 3D-printed using a specific biocompatible material. Personalized fixation plates and occlusal splints provide significant benefits, including enhanced precision and improved surgical stability [17,52,53]. Beyond mechanical accuracy and stability, the clinical reliability of digitally designed and manufactured surgical components also depends on their material properties and biocompatibility. Clinical studies evaluating CAD/CAM-fabricated cobalt–chromium alloys have demonstrated stable corrosion resistance and only transient ion release without inducing oxidative stress, supporting their safe use in digitally planned and manufactured maxillofacial applications [55].

Virtual osteotomy is performed in the mandible using Bilateral Sagittal Split Osteotomy (BSSO) and in the maxilla at Le Fort 1 level, separating the movable alveolar and palatal portion of the maxilla from the midface. The software then generates five segments: the midface, maxilla, corpus mandibulae, left and right ramus mandibulae [18]. Orthognathic surgery, whether for a single or both jaws, can be planned with digitally designed and 3D-printed intermediate and final splints, as well as cutting and positioning guides for procedures like genioplasty. A key benefit of virtual planning is its ability to model the changes in skeletal structure and predict how these alterations will affect the patient’s soft tissue, offering insights into the potential results [15,33,56].

Complications, such as excessive blood loss or nerve damage, can be minimized and surgical precision improved, as AI supports intraoperative decision-making by providing real-time insights and recommendations during surgical procedures [9,21,23,24].

#### 4.1.4. Outcome Prediction

Postoperative complications, treatment effectiveness and long-term outcomes can be predicted by AI models trained on large clinical and imaging datasets, which help doctors identify potential risks and support timely clinical decision-making, including early interventions and personalized follow-up care. It can also support clinical workflows by assisting in the analysis of radiographic images and other patient data. AI may also contribute indirectly to improved treatment outcomes by enhancing patient safety through early detection of risk factors and by predicting potential complications before they occur. AI-based applications can further support both safety and effectiveness by assisting doctors, providing predictive insights, and ensuring patients remain well-informed and protected throughout their treatment process [9,18,24,36,56,57,58].

### 4.2. Overview of the Included Literature and Quality Assessment

The reviewed literature demonstrated substantial heterogeneity in both methodological quality and research design. The majority of included publications consisted of narrative or scoping reviews, retrospective case series, and experimental or technical feasibility studies, reflecting the exploratory nature of current research in this field [4,23,27,43,59]. Only a limited number of studies employed a prospective design, and none reported randomized controlled trial (RCT) methodology [60]. In line with the scoping review methodology, no formal quality appraisal or evidence grading (e.g., OCEMB levels) was performed; instead, the included literature was descriptively synthesized to highlight recurring methodological characteristics and commonly reported limitations.

This distribution indicates that research on artificial intelligence (AI) in orthognathic surgery remains at an early developmental stage, with most studies focusing on proof-of-concept applications, technical feasibility, or descriptive outcomes rather than robust clinical validation [51,59,61].

Selection bias was prevalent, particularly among retrospective studies, which frequently lacked randomization or clearly defined inclusion and exclusion criteria. A substantial proportion of these studies relied on convenience samples or insufficiently described patient populations, as illustrated by works such as Yan et al. (2021) [36], Miragall et al. (2023) [23], and Kato et al. (2023) [33]. These limitations reduce internal validity and limit the reproducibility of reported findings.

Reporting transparency represented another major methodological concern. A considerable number of studies provided incomplete descriptions of their AI methodologies, often omitting essential details regarding model architecture, dataset size and composition, preprocessing procedures, or validation strategies. This issue was particularly evident in studies focusing on AI-assisted diagnosis or treatment planning, including those by Kato et al. (2023) [33], Du et al. (2024) [40], and Yan et al. (2021) [36]. Insufficient reporting hinders reproducibility and complicates meaningful comparison between studies.

External validity was limited, as only a small number of studies evaluated AI systems using independent or multi-institutional datasets. Notable exceptions include investigations by Abdul et al. (2024) [38], Rasteau et al. (2022) [27], and Farhud and Zokaei (2021) [26]. In many studies, models were developed using single-center datasets or retrospective cohorts, which may limit the generalizability of the reported outcomes to broader and more diverse clinical populations [33,36,40].

Bias management and algorithmic fairness were infrequently addressed across the included literature. Only a few studies explicitly discussed demographic representation, data imbalance, or potential sources of algorithmic bias in training datasets. Although ethical and conceptual concerns related to AI implementation were highlighted in narrative and ethical reviews [9,25,26], empirical evaluation of fairness or bias mitigation strategies was largely absent from primary research studies.

Overall, the methodological quality of the available literature reflects the early phase of AI integration into orthognathic surgery. Future research should prioritize well-designed prospective and multicentre clinical studies, standardized reporting frameworks, robust external validation, and transparent documentation of AI development and evaluation processes. Addressing these methodological gaps will be essential for strengthening scientific rigor and supporting the safe and effective clinical translation of AI-based technologies in orthognathic surgery.

### 4.3. Benefits of AI

The included studies highlight several areas where artificial intelligence (AI) is being explored for potential benefits in orthognathic surgery. Most commonly, AI has been applied to improve the accuracy and efficiency of cephalometric analysis. Several studies have shown that AI-based systems can reduce the time required for landmark identification and may decrease inter-observer variability [10,11,12,13,14]. Du [40] and Yan et al. [36] discussed the broader role of AI-assisted image analysis and decision-support tools in orthodontic and orthognathic diagnostics.

Virtual surgical planning (VSP) is another domain with frequent AI integration. Kato et al. [33] described AI-supported tools that facilitate 3D modelling, automated splint design, and soft tissue simulation, which may contribute to faster planning and increased predictability.

A smaller number of studies have explored the potential use of AI in intraoperative support. For instance, Zhang et al. [24] describe the integration of artificial intelligence in surgical workflows, highlighting its potential role in intraoperative decision support. Miragall et al. [23] discuss AI applications that could assist during surgical procedures, although such systems remain in early stages of development.

In the postoperative phase, machine learning models have been explored for predicting treatment outcomes, including relapse risk and patient satisfaction. For example, de Oliveira et al. [19] investigated the use of artificial intelligence as a prediction tool in orthognathic surgery assessment. Emmerling [18] and Bansal [56] further describe the broader role of digital AI-assisted tools in orthognathic planning and postoperative evaluation. Additionally, Rokhshad et al. [9] proposed AI as a tool in surgical education, emphasizing its potential to support personalized learning environments.

The reviewed studies show that AI is being used across multiple stages of the orthognathic workflow, with a focus on improving efficiency, standardization, and individualized planning. However, the overall clinical impact of these technologies still requires further validation.

### 4.4. Limitations and Challenges of AI Implementation in Orthognathic Surgery

Although artificial intelligence (AI) may improve the accuracy, personalization, and efficiency of orthognathic surgery, its integration into clinical practice still faces several limitations. These limitations span technical, clinical, and ethical domains, many of which are underrepresented or insufficiently addressed in the current literature.

#### 4.4.1. Technical Limitations

The foremost technical limitation is the lack of standardized datasets and annotation protocols, which hampers algorithm development and reproducibility. Current datasets vary in terms of image resolution, acquisition protocols, and cephalometric reference point definitions, resulting in limited cross-study comparability, and hindering model generalizability [36,40]. Deep learning models, despite their accuracy in detecting skeletal landmarks and planning virtual surgeries, are often trained on small, homogeneous samples, reducing their robustness across diverse populations [10].

Furthermore, many AI systems operate as “black boxes,” providing outputs without transparent explanations. This lack of interpretability complicates clinical validation and undermines trust in AI-generated recommendations, particularly when applied to high-stakes surgical decisions [23].

#### 4.4.2. Clinical Limitations

Clinically, over-reliance on AI can lead to diminished critical thinking and reduced situational awareness among surgeons. While AI aids in diagnostic imaging, virtual surgical planning (VSP), and splint design, surgeons must remain vigilant and able to override AI-generated plans in atypical or complex cases [23]. Overdependence risks deskilling and passive acceptance of suboptimal decisions.

Another important aspect is that AI tools are often not fully integrated with existing clinical infrastructure. Limited interoperability with electronic health records, CBCT software, and intraoperative tools decreases workflow efficiency. The lack of user-friendly interfaces and formal clinician training further impedes real-world adoption [18,33].

To overcome these barriers, future AI systems should prioritize seamless integration into clinical workflows and interoperability with existing technologies, such as CBCT and intraoral scanners. Equally important is structured user training, enabling clinicians to confidently and responsibly use AI-based tools while maintaining professional oversight and decision-making autonomy [62].

#### 4.4.3. Ethical, Legal, and Security Considerations

The use of AI technologies in surgical settings raises ethical, legal, and security questions that demand thorough investigation.

Ethical concerns primarily revolve around informed consent, algorithmic transparency, and equitable care. Patients may not fully understand the extent to which AI is involved in their diagnosis or treatment planning, challenging the ethical principle of autonomy [9]. It is essential that patients are adequately informed about AI’s role in their care, and that clinicians clearly communicate the nature and limitations of algorithmic support.

Another pressing issue is algorithmic bias. AI models trained on non-representative or biased datasets risk perpetuating healthcare disparities, particularly among underrepresented ethnic or socioeconomic groups [27,30]. Ensuring fairness and inclusivity in AI development and deployment is therefore a core ethical responsibility.

From a legal perspective, accountability for AI-driven decisions remains largely undefined. Under the current legal framework, the clinician retains primary responsibility for verifying and approving AI-generated recommendations before implementation. The AI system serves solely as a decision-support tool, and its output must always be interpreted by a qualified professional. However, as AI systems become increasingly autonomous, questions may arise regarding shared liability between clinicians, software developers, and healthcare institutions, particularly in cases involving algorithmic malfunction or insufficient software validation [29]. This legal ambiguity complicates malpractice litigation and highlights the importance of clear regulatory frameworks.

Data security and privacy are also critical issues. AI systems process highly sensitive personal health information, including 3D facial scans and radiographic data, making them attractive targets for cyberattacks. Unclear policies on data sharing, storage, and secondary use raise concerns regarding patient confidentiality and trust [26,38]. Robust cybersecurity protocols and transparent data governance are essential to safeguard patient rights.

As AI systems become increasingly autonomous, regulatory bodies must develop comprehensive guidelines to ensure ethical, safe, and accountable use in surgical care. This includes standards for algorithm validation, documentation, patient notification, and clinician oversight [25].

### 4.5. Gaps in the Current Literature

The current body of literature lacks large-scale, prospective, and multicentre studies that systematically evaluate AI tools in orthognathic surgery. Most evidence is derived from retrospective or experimental studies, limiting the strength and generalizability of conclusions [62].

Furthermore, there is a strong need for continuous real-world validation of AI algorithms. Mechanisms such as audit trails, performance monitoring, and feedback loops could help ensure long-term reliability, safety, and adaptability of AI systems in clinical environments [36,40].

Comparative studies assessing AI-assisted versus conventional surgical planning in terms of patient outcomes, surgical time, complication rates, and cost-effectiveness are especially scarce. Moreover, there is a need for studies focusing on long-term impacts, such as AI’s effect on surgical training, clinical decision-making, and patient–physician dynamics.

### 4.6. Interdisciplinary Collaboration

The development and successful implementation of AI tools in orthognathic surgery require close collaboration among clinicians, data scientists, engineers, and ethicists. Orthodontists and surgeons provide clinical expertise to define relevant anatomical parameters and treatment goals, while data scientists and engineers ensure algorithmic accuracy, transparency, and technical robustness. Ethical experts contribute by addressing issues of bias, accountability, and the patient consent.

Such interdisciplinary cooperation promotes not only clinically relevant and safe AI systems but also fosters innovation that aligns with real-world clinical needs. Establishing multidisciplinary teams and shared research infrastructures could therefore accelerate responsible AI adoption in surgical planning and patient care [9,14].

## 5. Conclusions

AI may play an important role in orthognathic surgery, particularly in areas such as diagnostic accuracy, surgical planning, and prediction of postoperative outcomes, thereby contributing to improved patient care and safety. The integration of AI in imaging, virtual surgical planning, and real-time decision-making demonstrates substantial benefits in precision and efficiency. However, challenges such as over-dependence on AI, privacy and security concerns, and ethical dilemmas regarding patient consent and accountability must be addressed to ensure responsible implementation. By balancing the strengths of AI with human expertise, healthcare providers can leverage AI technologies to improve surgical outcomes while safeguarding patient rights and maintaining trust in the healthcare system. Ethical considerations, strong data protection measures, and clear policies on AI usage are essential to ensure the responsible and effective adoption of AI in orthognathic surgery.

To facilitate the responsible translation of artificial intelligence into routine clinical orthognathic practice, future research should place greater emphasis on methodological transparency and external validation. Following established reporting guidelines for prediction models, such as TRIPOD, may improve the consistency and transparency of AI-based studies. In addition, the development of standardized and openly accessible datasets specific to orthognathic surgery could support reproducibility, benchmarking, and more robust comparison of AI-driven approaches across institutions.

## Figures and Tables

**Table 1:** Summary of included studies by application area of artificial intelligence in orthognathic surgery.

Application Area	Total Studies (*n*)	Study Type	Key Findings/Observations	Representative References
Cephalometric Analysis & Landmark Detection	13	Retrospective: 9, Experimental/Technical: 2, Review: 2	AI significantly reduces time and inter-observer variability in cephalometric landmark identification. Deep learning models demonstrate high accuracy in 2D cephalometry, while performance in 3D CBCT remains more variable.	[10,11,12,13,14]
Virtual Surgical Planning (VSP) & 3D Modelling	15	Retrospective: 6, Prospective: 1, Experimental/Technical: 5, Review: 3	AI-assisted VSP systems enables advanced 3D modelling, automated splint design, and soft tissue simulation, improving surgical planning efficiency and predictability.	[15,16,17,18]
Outcome Prediction & Postoperative Planning	10	Retrospective: 7, Prospective: 1, Review: 2	Machine learning models show potential in predicting relapse risk, postoperative outcomes, facial changes, and patient satisfaction; however, external validation and multicentre datasets are limited.	[19,20,21,22]
Intraoperative Assistance & Decision Support	6	Experimental/Technical: 4, Review: 2	Early AI-based systems may support intraoperative decision-making and workflow optimization, though most applications remain experimental and under development.	[2,23,24]
Ethics, Policy, Data Security & Bias	9	Conceptual/Review: 9	Studies emphasize ethical responsibility, data privacy, algorithmic bias, explainability, and the urgent need for regulatory and governance frameworks in medical AI.	[9,25,26,27]
General AI Reviews/Technical & Conceptual	9	Review: 9	Provides foundational and cross-disciplinary perspectives on AI principles, technical feasibility, limitations, and future directions, without direct clinical outcome evaluation.	[1,3,28,29,30]

## Data Availability

No new data was created or analysed in this study.

## Supplementary Materials

The following supporting information can be downloaded at: https://www.mdpi.com/article/10.3390/dj14050286/s1, Supplementary Table S1. Detailed characteristics of included primary studies applying artificial intelligence in orthognathic surgery and related fields, including study design, clinical application, artificial intelligence algorithms, datasets, training and validation strategies, and reported performance metrics.

## References

[1] Seo H.J., Choi Y.K. (2021). Current Trends in Orthognathic Surgery. Arch. Craniofac. Surg..

[2] Alten A., Gündeş E., Tuncer E., Kozanoğlu E., Akalın B.E., Emekli U. (2023). Integrating Artificial Intelligence in Orthognathic Surgery: A Case Study of ChatGPT’s Role in Enhancing Physician-Patient Consultations for Dentofacial Deformities. J. Plast. Reconstr. Aesthet. Surg..

[3] Kaplan A., Haenlein M. (2019). Siri, Siri, in My Hand: Who’s the Fairest in the Land? On the Interpretations, Illustrations, and Implications of Artificial Intelligence. Bus. Horiz..

[4] Khanagar S.B., Alfouzan K., Awawdeh M., Alkadi L., Albalawi F., Alghilan M.A. (2022). Performance of Artificial Intelligence Models Designed for Diagnosis, Treatment Planning and Predicting Prognosis of Orthognathic Surgery (OGS)—A Scoping Review. Appl. Sci..

[5] Tomášik J., Zsoldos M., Oravcová Ľ., Lifková M., Pavleová G., Strunga M., Thurzo A. (2024). AI and Face-Driven Orthodontics: A Scoping Review of Digital Advances in Diagnosis and Treatment Planning. AI.

[6] Tahir K., Abul Barakaat A., Fida M., Sukhia R.H. (2024). In the Contemporary Era of Artificial Intelligence, the Trajectory of Orthodontics: Past and Future Perspectives–A Narrative Review. J. Calif. Dent. Assoc..

[7] Strunga M., Ballová D.S., Tomášik J., Oravcová Ľ., Danišovič Ľ., Thurzo A. (2024). AI-Automated Cephalometric Tracing: A New Normal in Orthodontics?. Proceedings of the 2024 International Conference on Artificial Intelligence, Computer, Data Sciences and Applications (ACDSA).

[8] Tomášik J., Zsoldos M., Majdáková K., Fleischmann A., Oravcová Ľ., Sónak Ballová D., Thurzo A. (2024). The Potential of AI-Powered Face Enhancement Technologies in Face-Driven Orthodontic Treatment Planning. Appl. Sci..

[9] Rokhshad R., Keyhan S.O., Yousefi P. (2023). Artificial Intelligence Applications and Ethical Challenges in Oral and Maxillo-Facial Cosmetic Surgery: A Narrative Review. Maxillofac. Plast. Reconstr. Surg..

[10] Jiang F., Guo Y., Yang C., Zhou Y., Lin Y., Cheng F., Quan S., Feng Q., Li J. (2023). Artificial Intelligence System for Automated Landmark Localization and Analysis of Cephalometry. Dentomaxillofac. Radiol..

[11] Park J.H., Hwang H.W., Moon J.H., Yu Y., Kim H., Her S.B., Srinivasan G., Aljanabi M.N.A., Donatelli R.E., Lee S.J. (2019). Automated Identification of Cephalometric Landmarks: Part 1—Comparisons between the Latest Deep-Learning Methods YOLOV3 and SSD. Angle Orthod..

[12] Silva T.P., Hughes M.M., dos Santos Menezes L., de Melo M.d.F.B., de Freitas P.H.L., Takeshita W.M. (2022). Artificial Intelligence-Based Cephalometric Landmark Annotation and Measurements According to Arnett’s Analysis: Can We Trust a Bot to Do That?. Dentomaxillofac. Radiol..

[13] Kim H., Shim E., Park J., Kim Y.J., Lee U., Kim Y. (2020). Web-Based Fully Automated Cephalometric Analysis by Deep Learning. Comput. Methods Programs Biomed..

[14] Hwang H.W., Park J.H., Moon J.H., Yu Y., Kim H., Her S.B., Srinivasan G., Aljanabi M.N.A., Donatelli R.E., Lee S.J. (2020). Automated Identification of Cephalometric Landmarks: Part 2-Might It Be Better than Human?. Angle Orthod..

[15] Carrao V., Tofigh M., Greenberg A.M. (2018). Virtual Surgical Planning for Orthognathic Surgery. Digital Technologies in Craniomaxillofacial Surgery.

[16] Chin S.-J., Wilde F., Neuhaus M., Schramm A., Gellrich N.-C., Rana M. (2017). Accuracy of Virtual Surgical Planning of Orthognathic Surgery with Aid of CAD/CAM Fabricated Surgical Splint—A Novel 3D Analyzing Algorithm. J. Cranio-Maxillof. Surg..

[17] Lin H.H., Lonic D., Lo L.J. (2018). 3D Printing in Orthognathic Surgery—A Literature Review. J. Formos. Med. Assoc..

[18] Emmerling M.R., Shah B., Ginzburg M. (2024). Virtual Surgical Planning in Orthognathic Surgery. Curr. Surg. Rep..

[19] de Oliveira P.H.J., Li T., Li H., Gonçalves J.R., Santos-Pinto A., Gandini L.G., Cevidanes L.S., Toyama C., Feltrin G.P., Campanha A.A. (2024). Artificial Intelligence as a Prediction Tool for Orthognathic Surgery Assessment. Orthod. Craniofac. Res..

[20] Almarhoumi A.A. (2024). Accuracy of Artificial Intelligence in Predicting Facial Changes Post-Orthognathic Surgery: A Comprehensive Scoping Review. J. Clin. Exp. Dent..

[21] Stehrer R., Hingsammer L., Staudigl C., Hunger S., Malek M., Jacob M., Meier J. (2019). Machine Learning Based Prediction of Perioperative Blood Loss in Orthognathic Surgery. J. Cranio-Maxillofac. Surg..

[22] Shin W.S., Yeom H.G., Lee G.H., Yun J.P., Jeong S.H., Lee J.H., Kim H.K., Kim B.C. (2021). Deep Learning Based Prediction of Necessity for Orthognathic Surgery of Skeletal Malocclusion Using Cephalogram in Korean Individuals. BMC Oral Health.

[23] Miragall M.F., Knoedler S., Kauke-Navarro M., Saadoun R., Grabenhorst A., Grill F.D., Ritschl L.M., Fichter A.M., Safi A.F., Knoedler L. (2023). Face the Future—Artificial Intelligence in Oral and Maxillofacial Surgery. J. Clin. Med..

[24] Zhang C., Hallbeck M.S., Salehinejad H., Thiels C. (2024). The Integration of Artificial Intelligence in Robotic Surgery: A Narrative Review. Surgery.

[25] Thurzo A., Thurzo V. (2025). Embedding Fear in Medical AI: A Risk-Averse Framework for Safety and Ethics. AI.

[26] Farhud D.D., Zokaei S. (2021). Ethical Issues of Artificial Intelligence in Medicine and Healthcare. Iran. J. Public Health.

[27] Rasteau S., Ernenwein D., Savoldelli C., Bouletreau P. (2022). Artificial Intelligence for Oral and Maxillo-Facial Surgery: A Narrative Review. J. Stomatol. Oral Maxillofac. Surg..

[28] Noorbakhsh-Sabet N., Zand R., Zhang Y., Abedi V. (2019). Artificial Intelligence Transforms the Future of Health Care. Am. J. Med..

[29] Van Booven D., Cheng-Bang C., Meenakshy M. (2025). Limitations of Artificial Intelligence in Healthcare. Artificial Intelligence in Urologic Malignancies.

[30] Bhandari M., Zeffiro T., Reddiboina M. (2020). Artificial Intelligence and Robotic Surgery: Current Perspective and Future Directions. Curr. Opin. Urol..

[31] Muthuswamy Pandian S., Gandedkar N.H., kumar Palani S., Kim Y.J., Adel S.M. (2022). An Integrated 3D-Driven Protocol for Surgery First Orthognathic Approach (SFOA) Using Virtual Surgical Planning (VSP). Semin. Orthod..

[32] Mohammad-Rahimi H., Nadimi M., Rohban M.H., Shamsoddin E., Lee V.Y., Motamedian S.R. (2021). Machine Learning and Orthodontics, Current Trends and the Future Opportunities: A Scoping Review. Am. J. Orthod. Dentofac. Orthop..

[33] Kato R.M., Parizotto J.d.O.L., Oliveira P.H.J.d., Gonçalves J.R. (2023). Artificial Intelligence in Orthognathic Surgery—A Narrative Review of Surgical Digital Tools and 3D Orthognathic Surgical Planning. J. Calif. Dent. Assoc..

[34] Posnick J.C. (2021). Orthognathic Surgery: Past—Present—Future. J. Oral Maxillofac. Surg..

[35] Barone S., Antonelli A., Salviati M., Greco V., Bennardo F., Becker K., Giudice A., Simeone M. (2024). Accuracy Assessment of EM3D App-Based 3D Facial Scanning Compared to Cone Beam Computed Tomography. Dent. J..

[36] Yan K.-X., Liu L., Li H. (2021). Application of Machine Learning in Oral and Maxillofacial Surgery. Artif. Intell. Med. Imaging.

[37] Patil S., Albogami S., Hosmani J., Mujoo S., Kamil M.A., Mansour M.A., Abdul H.N., Bhandi S., Ahmed S.S.S.J. (2022). Artificial Intelligence in the Diagnosis of Oral Diseases: Applications and Pitfalls. Diagnostics.

[38] Abdul N.S., Shivakumar G.C., Sangappa S.B., Di Blasio M., Crimi S., Cicciù M., Minervini G. (2024). Applications of Artificial Intelligence in the Field of Oral and Maxillofacial Pathology: A Systematic Review and Meta-Analysis. BMC Oral Health.

[39] Vinayahalingam S., Berends B., Baan F., Moin D.A., van Luijn R., Bergé S., Xi T. (2023). Deep Learning for Automated Segmentation of the Temporomandibular Joint. J. Dent..

[40] Du W., Bi W., Liu Y., Zhu Z., Tai Y., Luo E. (2024). Machine Learning-Based Decision Support System for Orthognathic Diagnosis and Treatment Planning. BMC Oral Health.

[41] Liu J., Zhang C., Shan Z. (2023). Application of Artificial Intelligence in Orthodontics: Current State and Future Perspectives. Healthcare.

[42] Tanikawa C., Yamashiro T. (2021). Development of Novel Artificial Intelligence Systems to Predict Facial Morphology after Orthognathic Surgery and Orthodontic Treatment in Japanese Patients. Sci. Rep..

[43] Wong K.F., Lam X.Y., Jiang Y., Yeung A.W.K., Lin Y. (2023). Artificial Intelligence in Orthodontics and Orthognathic Surgery: A Bibliometric Analysis of the 100 Most-Cited Articles. Head Face Med..

[44] Hwang J.J., Jung Y.H., Cho B.H., Heo M.S. (2019). An Overview of Deep Learning in the Field of Dentistry. Imaging Sci. Dent..

[45] Lee K.S., Ryu J.J., Jang H.S., Lee D.Y., Jung S.K. (2020). Deep Convolutional Neural Networks Based Analysis of Cephalometric Radiographs for Differential Diagnosis of Orthognathic Surgery Indications. Appl. Sci..

[46] Jeong S.H., Yun J.P., Yeom H.G., Lim H.J., Lee J., Kim B.C. (2020). Deep Learning Based Discrimination of Soft Tissue Profiles Requiring Orthognathic Surgery by Facial Photographs. Sci. Rep..

[47] Albalawi F., Abalkhail K.A. (2022). Trends and Application of Artificial Intelligence Technology in Orthodontic Diagnosis and Treatment Planning—A Review. Appl. Sci..

[48] Vaira L.A., Biglio A., Favro A., Salzano G., Abbate V., Lechien J.R., De Riu G. (2024). Implant-Prosthetic Rehabilitation of the Atrophic Posterior Mandible with Additively Manufactured Custom-Made Subperiosteal Implants: A Cohort Study. Int. J. Oral Maxillofac. Surg..

[49] Buduru S., Hafidi S., Almășan O., Manziuc M., Tăut M., Buduru R., Nechita V.-I., Kui A., Chisnoiu A., Bacali C. (2024). Digital Condylar Parameter Assessment Using Cadiax^®^ 2 and Modjaw^®^. Dent. J..

[50] Paľovčík M., Tomášik J., Zsoldos M., Thurzo A. (2024). 3D-Printed Accessories and Auxiliaries in Orthodontic Treatment. Appl. Sci..

[51] Conley R.S. (2022). Orthognathic Surgery Past, Present, and Future. Clin. Investig. Orthod..

[52] Tichá D., Tomášik J., Oravcová Ľ., Thurzo A. (2024). Three-Dimensionally-Printed Polymer and Composite Materials for Dental Applications with Focus on Orthodontics. Polymers.

[53] Lepišová M., Tomášik J., Oravcová Ľ., Thurzo A. (2025). Three-Dimensional-Printed Elements Based on Polymer and Composite Materials in Dentistry: A Narrative Review. Bratisl. Med. J..

[54] Sampaio-Fernandes M.A., Pinto R.J., Almeida P.R., Sampaio-Fernandes M.M., Silva Marques D.N., Figueiral M.H. (2024). Direct vs. Indirect Digital Implant Impressions: A Time and Cost Analysis. Dent. J..

[55] Tomova Z., Vlahova A., Zlatev S., Stoeva I., Tomov D., Davcheva D., Hadzhigaev V. (2023). Clinical Evaluation of Corrosion Resistance, Ion Release, and Biocompatibility of CoCr Alloy for Metal-Ceramic Restorations Produced by CAD/CAM Technologies. Dent. J..

[56] Bansal S., Managutti A., Babhulkar A., Patel N. (2024). Artificial Intelligence in Oral and Maxillofacial Surgery: A Road Ahead. J. Oral Med. Oral Surg. Oral Pathol. Oral Radiol..

[57] Hartoonian S., Hosseini M., Yousefi I., Mahdian M., Ghazizadeh Ahsaie M. (2024). Applications of Artificial Intelligence in Dentomaxillofacial Imaging: A Systematic Review. Oral Surg. Oral Med. Oral Pathol. Oral Radiol..

[58] Yurdakurban E., Topsakal K.G., Duran G.S. (2024). A Comparative Analysis of AI-Based Chatbots: Assessing Data Quality in Orthognathic Surgery Related Patient Information. J. Stomatol. Oral Maxillofac. Surg..

[59] Takeshita W.M., Silva T.P., de Souza L.L.T., Tenorio J.M. (2024). State of the Art and Prospects for Artificial Intelligence in Orthognathic Surgery: A Systematic Review with Meta-Analysis. J. Stomatol. Oral Maxillofac. Surg..

[60] Sillmann Y.M., Monteiro J.L.G.C., Eber P., Baggio A.M.P., Peacock Z.S., Guastaldi F.P.S. (2025). Empowering Surgeons: Will Artificial Intelligence Change Oral and Maxillofacial Surgery?. Int. J. Oral Maxillofac. Surg..

[61] Bouletreau P., Makaremi M., Ibrahim B., Louvrier A., Sigaux N. (2019). Artificial Intelligence: Applications in Orthognathic Surgery. J. Stomatol. Oral Maxillofac. Surg..

[62] Wang H., Minnema J., Batenburg K.J., Forouzanfar T., Hu F.J., Wu G. (2021). Multiclass CBCT Image Segmentation for Orthodontics with Deep Learning. J. Dent. Res..

