# Supplementary material for: The Role of Artificial Intelligence in Orthognathic Surgery: A Scoping Review
Source: Dent J (Basel). 2026 May 11;14(5):286. doi: 10.3390/dj14050286 (PMC13206008; doi:10.3390/dj14050286)
Supplement: Supplementary file 1 [file dentistry-14-00286-s001.zip › dentistry-4096661-supplementary.pdf]

Supplementary Table S1. Detailed characteristics of included primary studies applying artificial intelligence in orthognathic surgery and related fields, including study design, clinical application, artificial intelligence algorithms, datasets, training and validation strategies, and reported performance metrics.

Author (Year)	Study Design	Clinical Application	AI Algorithm / Model	Input Data	Sample Size	Training / Validation Strategy	Performance Metrics (as reported)
Park et al. (2019)	Retrospective	Automated cephalometric landmark detection	YOLOv3; SSD	Lateral cephalograms	1311 images	Train: 1028; Test: 283	YOLOv3 runtime 0.05 s/image vs SSD 2.89 s/image; higher successful detection rate (SDR) for YOLOv3
Hwang et al. (2020)	Retrospective	Comparison of AI vs human landmark detection	YOLOv3	Lateral cephalograms	1311 images	Same dataset as Park et al.	Mean AI–human error 1.46 ± 2.97 mm; human–human error 1.50 ± 1.48 mm
Kim et al. (2020)	Retrospective	Fully automated web-based cephalometric analysis	Stacked hourglass deep neural network	Lateral cephalograms	2075 images	Train/validation/test (exact split not reported)	Mean prediction error 1.37 ± 1.79 mm; SDR ≤2 mm: 81.35%; classification accuracy 88.43%
Jiang et al. (2023)	Retrospective	Automated cephalometric analysis and classification	Two-stage cascade CNN (CephNet)	Lateral cephalograms	9870 images (multi-center)	Train: 9611; Validation: subset; Test: 259	Mean landmark error 0.94 ± 0.74 mm; SDR ≤2 mm: 91.73%; classification accuracy 89.33%
Silva et al. (2022)	Validation study	AI-assisted cephalometric landmark annotation (Arnett analysis)	Not reported (commercial AI software: CEFBOT)	Lateral cephalograms	30 images	Not applicable	ICC >0.94 for 8/10 measurements; no significant difference vs human examiner
Shin et al. (2021)	Retrospective	Prediction of need for orthognathic surgery	CNN (ResNet-based architecture)	Lateral and posteroanterior cephalograms	840 patients	Train/Validation/Test ratio 4:1:5	Accuracy 0.954; sensitivity 0.844; specificity 0.993
de Oliveira et al. (2024)	Retrospective	Prediction of orthognathic surgery vs orthodontic treatment	Ensemble of 10 ML models (RF, XGBoost, SVM, etc.)	Lateral cephalograms	920 patients	Train: 552; Validation: 183; Test: 185	Test AUC 0.791; accuracy 0.707; F1-score 0.706
Stehrer et al. (2019)	Retrospective	Prediction of perioperative blood loss	Random forest	Clinical and surgical data	950 patients	Train: 80%; Test: 20%	Mean prediction error 7.4 ml; significant correlation (p < 0.001)
Du et al. (2024)	Retrospective	AI-assisted diagnosis and surgical planning	XGBoost + adaptive artificial bee colony optimization	CT, cephalometric and clinical data	574 patients	Not reported (training cohort described, split not specified)	Diagnostic accuracy >90%; AUC >0.88 across deformity types

Vinayahalingam et al. (2023)	Retrospective	Automated TMJ segmentation for surgical planning	Three-stage 3D U-Net deep learning model	CBCT scans	162 CBCT scans	Train: 80%; Validation: 15%; Test: 5%	IoU up to 0.955; AI segmentation time 3.6 s vs >6 min manual
Chin et al. (2017)	Retrospective	Accuracy assessment of virtual surgical planning	Not applicable (computer-assisted VSP, no ML training)	CBCT scans	10 patients	Not applicable	No statistically significant deviation between planned and postoperative outcomes
Tanikawa & Yamashiro (2021)	Retrospective	Prediction of 3D facial soft-tissue changes	Geometric morphometrics + deep neural network	Cephalograms and 3D facial scans	137 patients	11-fold cross-validation	Mean error 0.94 mm (surgery); 0.69 mm (extraction); 100% success <2 mm

Abbreviations: AI, artificial intelligence; CNN, convolutional neural network; CBCT, cone-beam computed tomography; SDR, successful detection rate; ICC, intraclass correlation coefficient; AUC, area under the curve; *Not reported* indicates data not specified in the original publication; *Not applicable* indicates studies without model training or algorithm development.
